# Supplementary figures and images for: PTEN negatively regulates the cell lineage progression from NG2+ glial progenitor to oligodendrocyte via mTOR-independent signaling
Source: eLife. 2018 Feb 20;7:e32021. doi: 10.7554/eLife.32021 (PMC5839742; doi:10.7554/eLife.32021)

## Slide 1
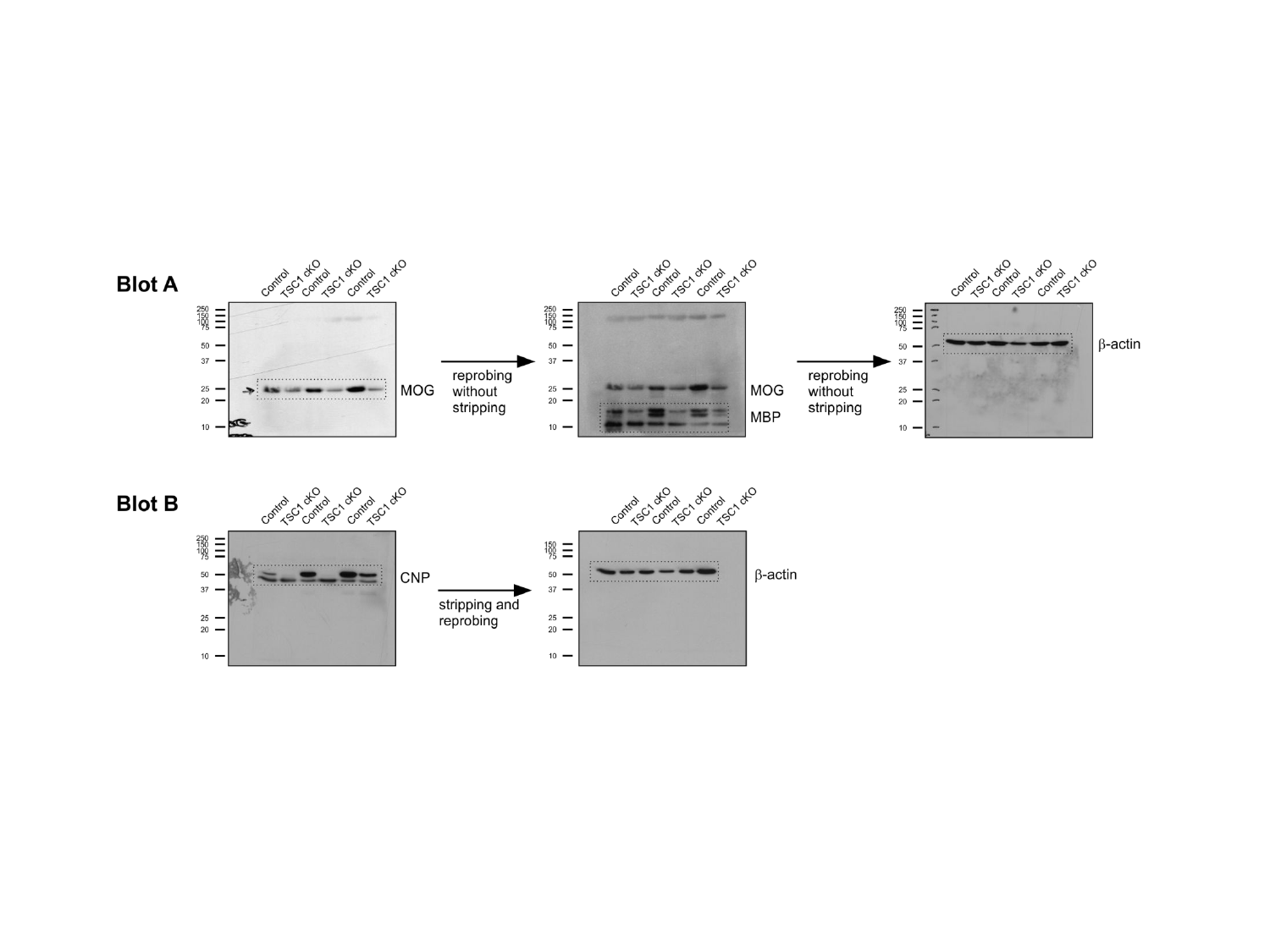

Supplement: Figure 1—source data 2. — Full-length western blot images from two separate blots (A and B). The blots were sequentially re-probed with the indicated Abs. The original images were cut with dashed-line boxes. [file elife-32021-fig1-data2.pptx]

## Slide 1
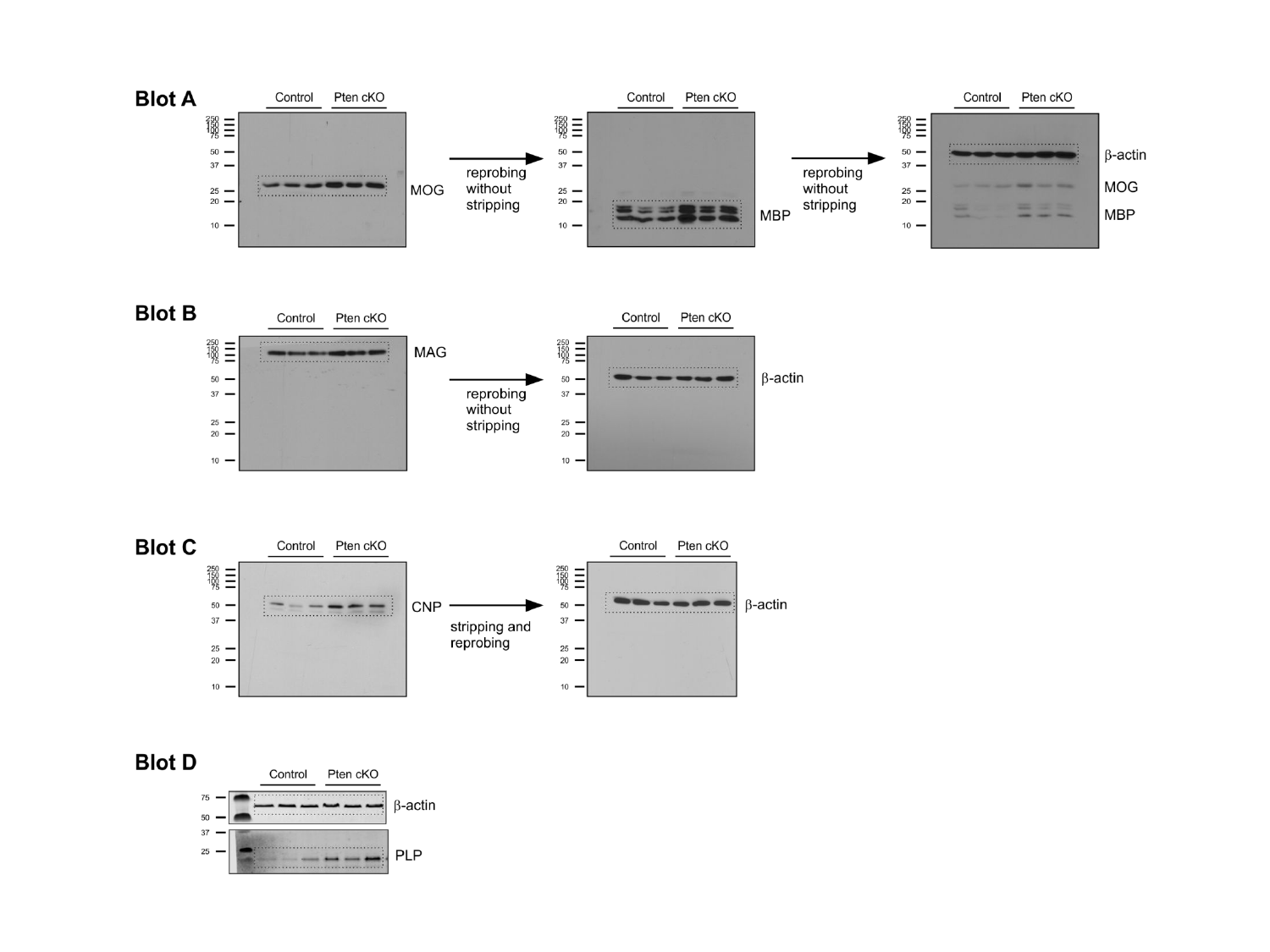

Supplement: Figure 4—source data 2. — Full-length western blot images from three separate blots (A, B, and C) used in Figure 4A. The blots were sequentially re-probed as indicated. The original images were cut with dashed-line boxes. The blot D was used for the simultaneous detection PLP and β-actin using Odyssey infrared scanner (LI-COR). [file elife-32021-fig4-data2.pptx]

## Slide 1
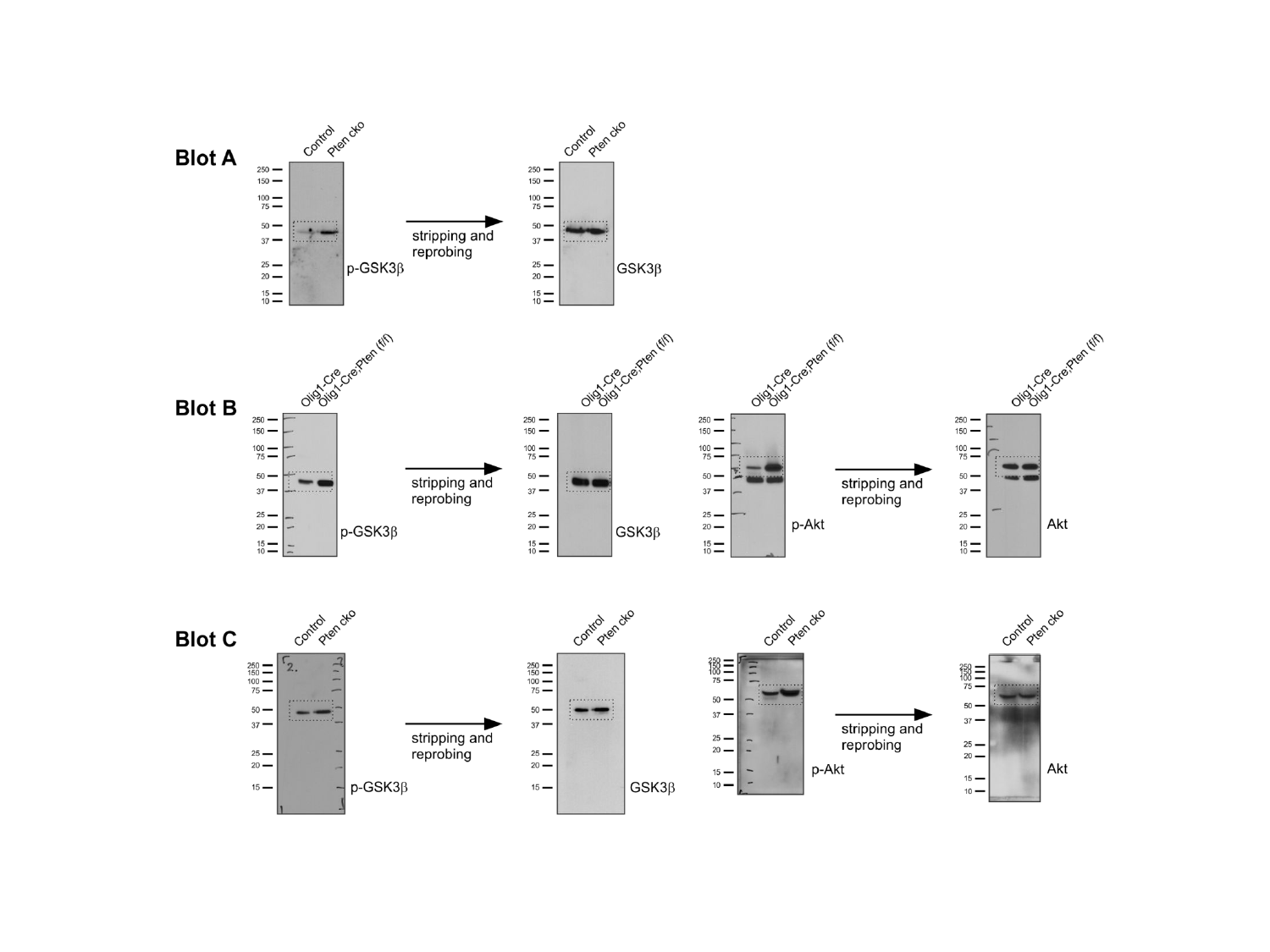

Supplement: Figure 8—source data 2. — Full-length western blot images from three separate blots (A, B, and C) used in Figure 8E–H. The blots were stripped and sequentially re-probed as indicated. The original images were cut with dashed-line boxes. [file elife-32021-fig8-data2.pptx]

## Slide 1
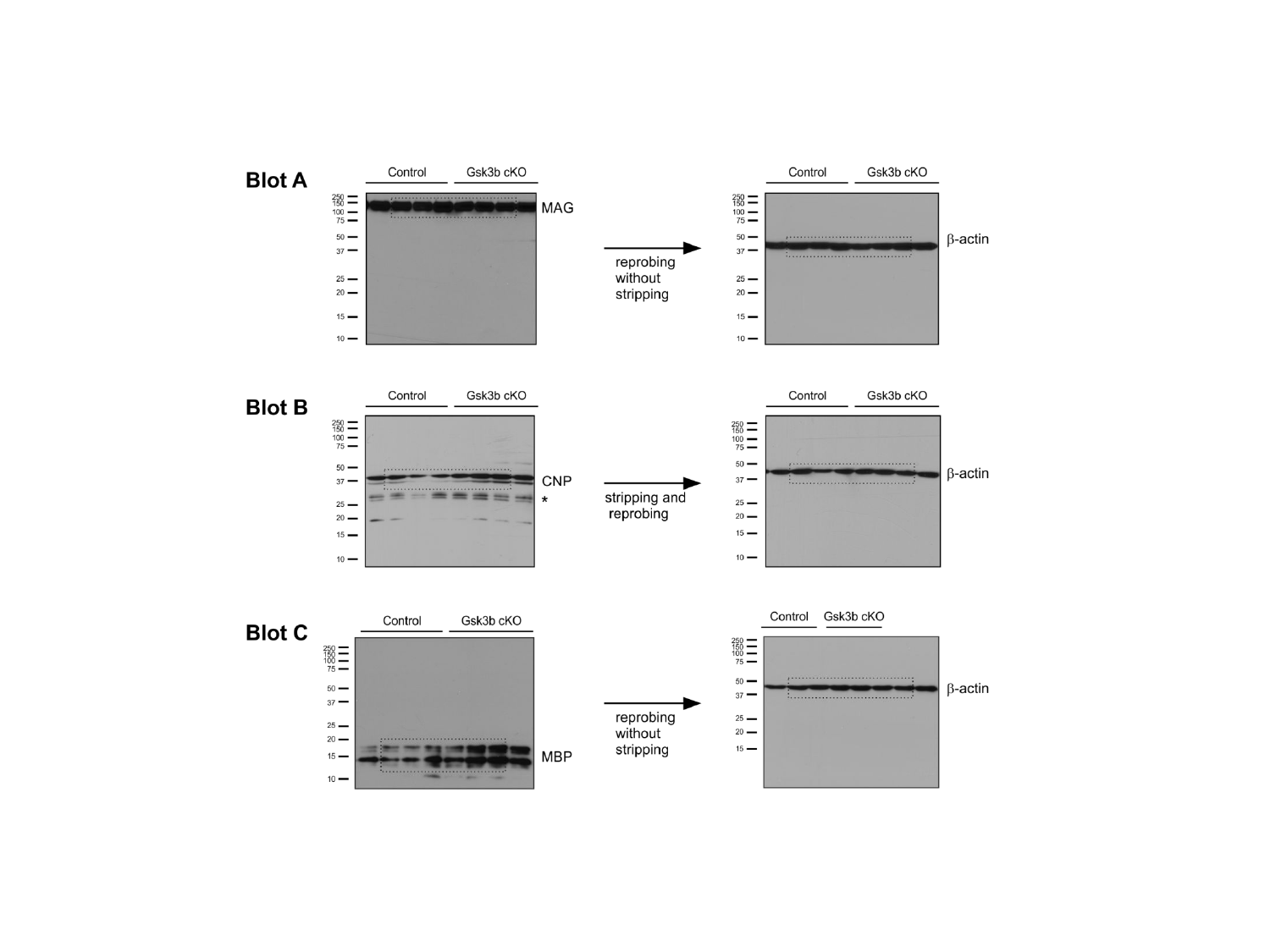

Supplement: Figure 9—figure supplement 1—source data 2. — Full-length western blot images from three separate blots (A, B, and C). The blots were re-probed with β-actin Ab as indicated. The original images were cut as indicated with dashed-line boxes. The asterisk indicates non-specific signals. [file elife-32021-fig9-figsupp1-data2.pptx]
